# 

**Published:** 1842-09

**Authors:** 


### CONTENTS.

### LIBRARY.

PAGE.

Principles of Dental Surgery; exhibiting a new method of treating the Diseases of the Teeth and Gums. By Leonard Koecker, Surgeon Dentist, London,........................ 169

### JOURNAL.

ARTICLE I.—Introductory Address. By Eleazar Parmly, M. D., D. D. S.,........................ 1

ARTICLE II.—Dissertation on the Diseases of the Maxillary Sinus. By Chapin A. Harris, M. D., D. D. S.,........................ 20

ARTICLE III.—A Remedy for the Protrusion of the Lower Jaw. By J. S. Gunnell, M. D., Washington City,........................ 65

### MISCELLANEOUS NOTICES.

American Society of Dental Surgeons,........................ 69
Catalogue of Officers and Members,........................
Introductory Address,........................ 75
To Correspondents,........................ 76
Artificial Teeth Worn by Washington,........................ 76
Essay on Salivary Calculus,........................ 77
Mechanical Dentistry,........................ 77
Dr. Wm. P. Greenwood and Mr. Peabody,........................ 77
Vascularity of Tooth-bone,........................ 78
Dissertation on the Methods of Degrading the Dental Profession,.... 78
Notice from new Treasurer,........................ 78
Boylston Medical Prize Questions,........................ 78
W. H. Elliot's Improved Method of Pivoting Teeth,........................ 80
Dr. Maynard's Drill Stock,........................ 80
Obituary—Anson B. Hayden, D. D. S........................ 80

